# Effects of a Paleolithic diet compared to a diabetes diet on leptin binding inhibition in secondary analysis of a randomised cross-over study

**DOI:** 10.1186/s12902-024-01715-0

**Published:** 2024-09-04

**Authors:** Maelán Fontes-Villalba, Yvonne Granfeldt, Kristina Sundquist, Ashfaque A. Memon, Anna Hedelius, Pedro Carrera-Bastos, Tommy Jönsson

**Affiliations:** 1 Center for Primary Health Care Research. Department of Clinical Sciences. Malmö, Lund University, Malmö, Sweden; 2 Deparment of Process and Life Science Engineering, Lund University, Lund, Sweden; 3 University Clinic Primary Care Skåne, Region Skåne, Malmö, Sweden; 4 Costa Teguise, Spain

**Keywords:** Paleolithic diet, Type 2 diabetes, Leptin, Leptin resistance, Wheat gluten, BioLep

## Abstract

**Background:**

Beneficial effects from practising a Paleolithic diet as compared to a diabetes diet on weight, waist circumference, satiety, leptin, HbA1c and glucose control in randomised controlled trial participants with type 2 diabetes could be due to lower leptin resistance. Support for this hypothesis comes from an in vitro experiment that showed that digested wheat gluten, which is excluded from a Paleolithic diet, inhibits leptin from binding to its receptor, thus indicating a possible dietary cause of leptin resistance. However, the clinical relevance of the latter finding is unclear since removal of enzyme activity from the gluten digest by heat treatment also abolished leptin binding inhibition. Assessment of leptin binding inhibition in vivo is possible by comparison of total leptin levels with those of ‘biologically active’ leptin bound to its receptor (bioLep).

**Objectives:**

To assess the effects of a Paleolithic diet compared to a diabetes diet on leptin binding inhibition and to replicate our in vitro study.

**Methods:**

BioLep and total leptin levels were measured in secondary analysis of fasting plasma samples from our open label random order three plus three-month long cross-over trial performed in 2005–2007, that compared a Paleolithic diet with a diabetes diet in participants with type 2 diabetes without insulin treatment (per protocol). BioLep was also measured in vitro for known recombinant leptin concentrations incubated with a series of concentrations of 10 kDa spin-filtered digested wheat gluten, with or without prior heat treatment, at 100ºC for 30 min and centrifugation.

**Results:**

There was no difference between diets when comparing differences between bioLep and total leptin levels and their ratio in the 13 participants, three women and 10 men, aged 52–74 years with a mean BMI of 30 kg/m^2^ and a mean diabetes duration of eight years. We found no carry-over or period effect for bioLep and total leptin. In vitro, wheat gluten digest inhibited leptin binding in a dose-dependent manner but not after heat treatment.

**Conclusions:**

We found no leptin binding inhibition after the Paleolithic or diabetes diet, possibly due to its abolishment from cooking-related heat treatment of wheat gluten.

**Trial registration:**

Registered on 14/02/2007 at ClinicalTrials.gov Identifier: NCT00435240.

## Background

Modern chronic diseases such as coronary heart disease, hypertension and diabetes appear to be absent among recent hunter-gatherer populations [1]. Based on this epidemiological observation, it has been proposed that the diet of recent hunter-gatherer populations may be ideal for helping prevent and treat modern chronic diseases such as coronary heart disease, hypertension, and diabetes [1, 2]. The suggested theoretical underpinning of the proposition is that modern chronic diseases arise due to insufficient genetic adaptation to a recently (in evolutionary terms) introduced agricultural diet [1, 3]. The diets of recent hunter-gatherer populations are thought to resemble most closely those of preagricultural human populations during the late Paleolithic period [1]. Therefore, the general dietary pattern of recent hunter-gatherer populations is now usually referred to as a “Paleolithic diet” and includes fruits and vegetables, roots and tubers, lean meats, fish, seafood, eggs and nuts, and excludes cereal grains, dairy products, legumes, refined fats and sugar [1–3]. The Paleolithic diet has been tested in randomised controlled intervention studies with encouraging results on risk factors for modern chronic diseases [4–7]. Our random order three plus three-month long cross-over diet intervention study in people affected by type 2 diabetes resulted in lower body weight, leptin and HbA1c, and higher satiety per calorie from practising the Paleolithic diet as compared to the diabetes diet [8–10]. A potential explanation for the observed beneficial effects could be a greater decrease in leptin resistance due to practising the Paleolithic diet [10].

Leptin is a hormone secreted by adipose tissue that promotes satiety and has a central role in energy balance and weight management. Leptin resistance is an unclearly defined and not fully understood state of impaired leptin function linked to food intake and is commonly seen in cases of obesity [11]. In light of the above, it is interesting that we in vitro have shown, using surface plasmon resonance technology, that wheat gluten (which is absent in the Paleolithic diet) digested with gut enzymes to mimic physiological conditions in the human intestine inhibits leptin from binding to the leptin receptor at clinically relevant concentrations, thus indicating a possible dietary cause of leptin resistance [12]. This finding, translated to the clinical setting of our cross-over study, should result in a difference in leptin binding inhibition between the wheat gluten-free Paleolithic diet and the wheat gluten-containing diabetes diet. However, the clinical relevance is unclear since the removal of enzyme activity from the gluten digest by heat treatment also abolished leptin binding inhibition in vitro [12]. Since wheat proteins are almost exclusively eaten after cooking, dietary leptin binding inhibition could in vivo be abolished or reduced. Thus, it is unclear if the in vitro finding translates to a dietary leptin binding inhibition in vivo. This uncertainty would be addressed by measurements of leptin binding inhibition in stored plasma samples from our cross-over study.

After the measurement of leptin in our clinical intervention study and our in vitro study on gluten digest and leptin binding inhibition, commercially available laboratory kits with the ability to measure biologically active leptin (bioLep) have become available [13]. The ‘biologically active’ aspect refers to the ability of the new kits to measure how much leptin binds to the leptin binding site of the leptin receptor [13]. Such measurements in combination with traditional measures of total leptin enable us to detect the relative amount of receptor-binding leptin and thereby also assess leptin binding inhibition. Our aim in this study was to assess the effect of a Paleolithic diet as compared to a diabetes diet on leptin binding inhibition by measurements of bioLep and total leptin in secondary analysis of stored plasma samples from our randomised cross-over trial. Another aim of this study was to replicate our in vitro surface plasmon resonance technology study using the bioLep laboratory kit.

## Methods

BioLep and total leptin were measured at the Center for Primary Health Care Research lab in Malmö using bioLEP ELISA L07 and LEP ELISA E07, respectively, from Mediagnost according to the manufacturer’s instructions. Each run included two controls of known concentration and a blank. The bioLEP ELISA L07 is a ligand-binding immunoassay for quantitative determination of bioLep. Briefly, recombinant extracellular domain of the human leptin receptor is immobilized on a microtiter plate. Added bioLep in gluten digest or plasma bioLep is bound to the immobilized receptor and subsequently detected by a highly specific polyclonal, biotin-conjugated anti-leptin antibody and a streptavidin-peroxidase conjugate. Non-receptor-binding leptin does not give any signal. The assay is performed according to the WHO International Standard for human leptin, NIBSC 97/594.

### In vivo study design

For this part of the study, we measured bioLep and total leptin per protocol after each diet in a secondary analysis of -80 °C stored fasting plasma samples from our random order three plus three month long cross-over diet intervention study, which was performed in 2005–2007 [8]. This study compared a Paleolithic diet, based on the general dietary pattern of recent hunter-gatherer populations [14] with a diabetes diet, based on the evidence-based nutritional approaches to the treatment and prevention of diabetes mellitus by the Diabetes and Nutrition Study Group (DNSG) of the European Association for the Study of Diabetes (EASD) [15], in individuals affected by type 2 diabetes [8]. For full details about the methods and previously reported results, please see [8–10, 16]. Approval of the study was obtained from the Regional Ethical Board in Lund (LU 726/2004 and 711/2013). All participants gave written informed consent.

Trial registered on 14/02/2007 at Clinicaltrials.gov (Identifier: NCT00435240).

#### Participants

Individuals affected by type 2 diabetes without insulin treatment were recruited from primary healthcare units in the Lund area of southern Sweden. Inclusion criteria were adult individuals affected by type 2 diabetes without insulin treatment and a C-peptide value above zero, unaltered medical diabetes treatment and stable weight since three months before the start of the study, HbA1c above 5.5% by Mono-S standard, creatinine below 130 μmol/L, liver enzymes below four times their respective upper reference value, no chronic oral or injection steroid treatment and no acute coronary event or change in medication of beta blockers or thyroxin since six months before the start of the study. Exclusion criteria were insulin treatment or chronic steroid treatment (not inhalation), warfarin treatment, creatinine above 130 μmol/L or liver enzymes above four times their respective upper reference value, acute coronary event, change in beta blocker or thyroxin medication, and physical or psychological illness or changes in personal circumstances that would make further study participation impossible.

#### Diets

The Paleolithic diet was based on lean meat, fish, fruit, vegetables, root vegetables, eggs and nuts, while avoiding dairy products, cereal grains, legumes, refined fats, sugar, candy, soft drinks, beer and added salt [14]. The diabetes diet was based on an increased intake of vegetables, root vegetables, dietary fibre, wholegrain bread and other wholegrain cereal products, fruits and berries, and decreased intake of total fat with more unsaturated fat [15]. The difference in cereal grain intake between the diets should lead to a corresponding difference in wheat gluten content.

#### Procedures

All eligible individuals were informed of the intention to compare two healthy diets and that it was not known whether one diet might be superior to the other. After randomisation was complete, there was no blinding of dietary assignment to study participants, nor to those administering the interventions or assessing the outcomes. Upon commencement of the study, all participants were randomised, by use of opaque envelopes, to start with either a diabetes diet designed in accordance with official recommendations or a Palaeolithic diet. Immediately after randomisation, all participants received individual oral and written information about their respective initial diet from diabetes nurses. After three months, all participants switched diets and received new oral and written information about the new diet. Advice about regular physical activity was given equally to all participants. Fasting venous blood samples were obtained in the morning, followed by an oral glucose tolerance test and measurements of blood pressure, weight and waist circumference using standard methods at the start of the study, after three months (when switching to a new diet) and at the end of the study (after six months). Samples were collected in EDTA-containing tubes and centrifuged at 1,700 g for 10 min at 4 °C. Plasma was then aliquoted and stored at − 80 °C until analysis. Food intake during each diet study was assessed from four-day weighed food records that started approximately six weeks after initiating each diet.

### In vitro study design

For this part of the study, we used -80° C stored 10 kDa spin-filtered gluten digest from our surface plasmon resonance technology study [12]. For details on gluten digest manufacture, please see [12]. Briefly, to mimic physiological conditions in the human intestine, gluten from wheat was digested with the gut enzymes pepsin and trypsin. Enzyme activity from pepsin and trypsin (molecular weight ~ 40 and 25 kDa, respectively) was subsequently removed from the gluten digest by either spin-filtering through a 10 kDa filter or heat treatment at 100ºC for 30 min followed by centrifugation at 13,000 g for 10 min.

#### Procedures

The gluten digest protein concentration was determined by Pierce BCA Protein Assay Kit (Thermo Scientific, Rockford, USA). The effects of heat treatment and centrifugation on gluten digest concentration were assessed by measuring the concentration of gluten digest before and after heat treatment at 100 °C for 30 min, heat treatment at 100 °C for 30 min followed by centrifugation at 13,000 g for 10 min, and only centrifugation at 13,000 g for 10 min without heat treatment.

BioLep was measured in triplets for recombinant leptin at 10 and 50 ng/mL concentrations (recombinant human leptin, R&D Systems 398-LP, the same as used in our in vitro study using surface plasmon resonance technology) [12], incubated with a series of increasing gluten digest concentrations ranging from 0 to 320 μg/mL. The recombinant leptin concentration of 10 ng/mL was chosen as a close representation of mean in vivo leptin concentrations in our randomised cross-over trial and the recombinant leptin concentration of 50 ng/mL was chosen as a representation of a much higher but still clinically plausible concentration, both of which are well within the detection limits of the laboratory kit. BioLep was also measured for recombinant leptin at 10 ng/mL concentration incubated with gluten digest concentrations ranging from 5 to 320 μg/mL after the gluten digest had been subjected to heat treatment at 100ºC for 30 min, heat treatment at 100ºC for 30 min followed by centrifugation at 13,000 g for 10 min or only centrifugation at 13,000 g for 10 min.

### Statistics

This is a secondary analysis of a study in which the small sample size reflects power calculations for previously reported cardiometabolic outcomes. The sample size was deemed sufficient for this study on leptin binding inhibition based on our previous finding of a half-maximal inhibition of leptin binding at a gluten digest concentration of 10 ng/mL, which is well below the 41 ng/mL mean concentration reported for undegraded gliadin in about half (14 of 31) of healthy adult human sera [17]. Gliadin is the prolamin protein of wheat and makes up about half of all wheat gluten. Assuming that the leptin binding inhibitory effect of gluten digest concentrations is at least roughly translatable to similar gliadin concentrations, a greater than half-maximal inhibition of leptin binding could be expected in about half of the samples. The study would then require a sample size of at least seven participants to achieve a power of 80% and a level of significance of 5% (two-sided), for detecting a mean of the differences of 0.25 between pairs, assuming a pooled standard deviation of 0.18 for the differences.

A two-sided paired-samples t-test or Wilcoxon matched-pairs signed rank sum test was used for group mean comparisons, as appropriate, and Spearman’s rank test was used for correlation. Statistical significance was set at *p* < 0.05. Carry-over effect was tested by comparing variable means of first and second diet for the group starting with the Palaeolithic diet with those for the group starting with the diabetes diet. Period effect was tested by comparing variable means between the first and second diets. Analysis was conducted by use of IBM SPSS Statistics for Macintosh, Version 28.

## Results

### Previously reported

A total of 17 individuals out of 26 assessed for eligibility were randomised and started the study [8]. Four participants were excluded for the following reasons: one starting with the Paleolithic diet was wrongly included due to ongoing warfarin treatment; one starting with the Paleolithic diet was unwilling to continue due to abdominal pains and bloating; one starting with the diabetes diet was excluded after developing leukaemia; and one starting with the diabetes diet was excluded after developing heart failure [8]. The 13 participants who completed the study and were analysed consisted of three women and 10 men aged 52–74 years with a mean BMI of 30 kg/m^2^ and a mean diabetes duration of eight years (baseline characteristics summarised in Table 1) [8]. Mean (SD) daily intake in grams of rice and other cereal grains were 7 (17) and 11 (24) grams for the Paleolithic diet and 6 (10) and 172 (96) grams for the diabetes diet, respectively [8]. The Paleolithic diet resulted in higher satiety, a more beneficial lipid profile, lower body weight, waist circumference, HbA1c and total leptin levels compared to the diabetes diet [8–10].

**Table 1:** Baseline Characteristics

Variable	Mean (SD)	
Sex male/female, n	10/3	
Age, year	64	(6)
Diabetes duration, year	8	(5)
HbA1C, %, Mono-S	6.6	(0.6)
fP-glucose, mmol/L	7.8	(1.2)
Cholesterol, mmol/L	4.4	(1.1)
Low-density lipoprotein cholesterol, mmol/L	2.9	(0.9)
High-density lipoprotein cholesterol, mmol/L	1.3	(0.2)
C-reactive protein, mg/L	2.4	(1.8)
Systolic blood pressure, mmHg	150	(21)
Diastolic blood pressure, mmHg	83	(6)
Height, cm	171	(5)
Weight, kg	87	(17)
Body mass index, kg/m^2^	30	(7)
Waist circumference, cm	103	(14)

### New measurements

Intra assay variance (coefficient of variability) of the bioLEP ELISA L07 and LEP ELISA E07 measurements were just below 5% with no inter assay variance since each run contained all measurements in one plate.

#### In vivo

There was no difference between bioLep and total leptin after the Paleolithic or diabetes diet and consequently also no difference between diets when comparing differences between bioLep and total leptin or their ratio (Table 2). Both bioLep and total leptin were lower after the Paleolithic diet compared to after the diabetes diet (Table 2). As stated in the background section, total leptin has been measured previously in these samples [10]. New and old measurements were highly correlated (rs = 0.97, *p* < 0.001) with new measurements being on average 67% higher compared to old measurements, most likely due to liquid evaporation during storage. We found no carry-over or period effect for bioLep and total leptin.

**Table 2:** BioLep and Total Leptin After the Paleolithic and Diabetes Diet

Variable	Paleolithic diet	Diabetes diet	*p*^a^
BioLep, ng/mL *M (SD)*	8.5 (6.0)	11.8 (9.3)	.02
Total leptin, ng/mL *M (SD)*	8.8 (6.7)	12.1 (9.9)	.02
*p*^b^	.2	.3	
BioLep minus total leptin, ng/mL *M (SD)*	-0.3 (0.8)	-0.3 (0.9)	.7
BioLep to total leptin ratio, *M (SD)*	0.96 (0.18)	1.02 (0.15)	.4

^a^*p* for mean comparison between diets. ^b^*p* for mean comparison between bioLep and total leptin for each diet

#### In vitro

**Fig. 1:**
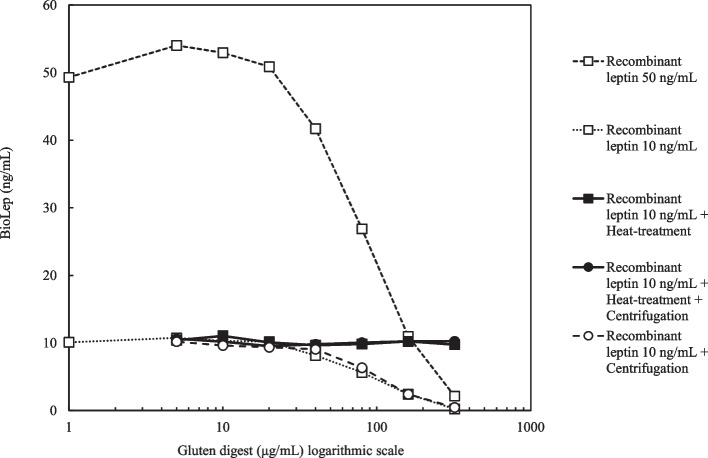
BioLep for Recombinant Leptin Incubated with a Series of Increasing Gluten Digest Concentrations. *Note.* * BioLep concentrations at 0 μg/mL gluten digest concentration is here presented at 1 μg/mL gluten digest concentration since the logarithm of 0 is undefined and cannot be presented on the x-axis

There was no apparent effect on gluten digest concentration (190 μg/mL) from heat treatment (192 μg/mL) and/or centrifugation (194 μg/mL and 196 μg/mL, respectively). Heat-treated gluten digest with or without centrifugation did not reduce bioLep (Fig. 1). Gluten digest with or without centrifugation reduced bioLep in a dose-dependent manner and at similar concentrations for both 10 and 50 ng/mL recombinant leptin (Fig. 1).

## Discussion

We found no difference between bioLep and total leptin after the Paleolithic or diabetes diet in a secondary analysis of stored plasma samples from our randomised cross-over trial, and consequently also no difference between the diets when comparing differences between bioLep and total leptin or their ratio. We thus found no leptin binding inhibition in vivo. Our results concur with the possibility that cooking-related heat treatment abolishes leptin binding inhibition caused by gluten digest. Other possibilities could be that gluten digest does not reach sufficient systemic concentration [18] or that leptin in vivo differs from its recombinant counterpart [19]. BioLep levels and bioLep to total leptin ratios in our study are in-line with previous results found in a clinical cohort of 409 lean and obese children and adults [13]. There were no dietary data for this cohort, but daily wheat gluten consumption was estimated in the Western diet thought to range from 5 to 20 g [20]. Based on estimated relative food supply quantities in Sweden in 2020 and assuming an average gluten content of wheat of around 8%, the cereal grain intake in this study translates into a mean daily wheat gluten intake of 1 g for the Paleolithic diet and 12 g for the diabetes diet [20, 21]. The daily wheat gluten intake of 12 g for the diabetes diet is well within the Western diet range but more than ten-fold higher compared to the wheat gluten intake of 1 g for the Paleolithic diet.

Furthermore, we found that gluten digest, with or without centrifugation, reduced bioLep in a dose-dependent manner similarly for both 10 and 50 ng/mL recombinant leptin. This indicates a dose-dependent leptin binding inhibition by gluten digest, which replicates our previous in vitro study findings [12]. The similar leptin binding inhibition at similar concentrations of gluten digest for both 10 ng/mL and 50 ng/mL recombinant leptin suggests that gluten digest inhibits the leptin receptor in a non-competitive way from binding to leptin. We also found that heat treatment of gluten digest abolished the leptin binding inhibition, which is in line with both our in vivo findings from this study and our previous in vitro study findings [12].

### Limitations of this study

Firstly, our results do not allow us to preclude the possibility that heat-treated digests of gluten, or other cereal grain proteins, can disturb leptin function other than leptin binding. Such a disturbance may affect intracellular signalling of the leptin receptor and that would still constitute a dietary cause of leptin resistance [22].

Secondly, since our in vivo results are from blood samples taken from participants in a fasting state, they do not allow us to preclude the possibility of transient meal effects on leptin binding. Kinetics for dietary antigens in healthy adults have been assessed for ovalbumin with levels rising from undetectable to peak levels of 1.7 to 10.5 ng/ml 2–3 h after a milk and egg test meal, and the maximal total amount of dietary antigen found in the circulation corresponded to 10^–5^ fraction of the amount consumed [23]. The 41 ng/mL mean concentration reported for undegraded gliadin in only about half (14 of 31) of healthy adult human sera indicates a possibly similar meal variability [17].

Thirdly, our in vitro studies with heat treatment after enzyme digestion of gluten is an order reversal of in vivo conditions. It is possible, albeit unlikely, that heat treatment before enzyme digestion of gluten would have produced different in vitro results.

## Conclusions

We found no leptin binding inhibition in vivo after the Paleolithic and the diabetes diet, which concurs with the possibility that cooking-related heat treatment abolishes leptin binding inhibition from gluten digest. Using another laboratory method, we also replicated our previous in vitro finding of a dose-dependent leptin binding inhibition from gluten digest that was abolished by heat treatment of the gluten digest.

## Data Availability

The dataset analysed during the current study is available from the corresponding author upon reasonable request.

## Abbreviations

BMI: Body mass index
HbA1c: Glycated haemoglobin
kDA: Kilodaltons
